# Macrophages and lipid metabolism

**DOI:** 10.1016/j.cellimm.2018.01.020

**Published:** 2018-08

**Authors:** Anneleen Remmerie, Charlotte L. Scott

**Affiliations:** a Laboratory of Myeloid Cell Ontogeny and Functional Specialization, VIB-UGent Center for Inflammation Research, Technologiepark 927, Ghent, Belgium; b Department of Biomedical Molecular Biology, Ghent University, Ghent, Belgium; c Institute of Infection, Immunity and Inflammation, College of Medical, Veterinary and Life Sciences, University of Glasgow, Glasgow, UK

**Keywords:** Macrophages, Lipid metabolism, NAFLD, PAP, Atherosclerosis, AMD

## Abstract

Distinct macrophage populations throughout the body display highly heterogeneous transcriptional and epigenetic programs. Recent research has highlighted that these profiles enable the different macrophage populations to perform distinct functions as required in their tissue of residence, in addition to the prototypical macrophage functions such as in innate immunity. These ‘extra’ tissue-specific functions have been termed accessory functions. One such putative accessory function is lipid metabolism, with macrophages in the lung and liver in particular being associated with this function. As it is now appreciated that cell metabolism not only provides energy but also greatly influences the phenotype and function of the cell, here we review how lipid metabolism affects macrophage phenotype and function and the specific roles played by macrophages in the pathogenesis of lipid-related diseases. In addition, we highlight the current questions limiting our understanding of the role of macrophages in lipid metabolism.

## Highlights

• The transcriptional signature of Kupffer cells & Alveolar macrophages are enriched for lipid metabolism genes.
• Lipid metabolism may control macrophage phenotype.
• Dysregulated lipid metabolism in macrophages contributes to disease pathology.

## 1 Introduction

Macrophages (Mϕs), first described over a century ago by Ellie Metchnikoff, are found in most tissues of the body. Originally proposed by Van Furth to be part of the mononuclear phagocyte system, originating from bone marrow (BM) derived monocytes [1], it is now clear that tissue-resident Mϕs are derived during embryogenesis from yolk-sac Mϕs and/or fetal liver monocytes [2], [3], [4], [5], [6]. However, in accessible tissues, such as the liver and spleen, BM monocytes can engraft and contribute to the tissue-resident Mϕ pool during both the neonatal window and in certain tissues, such as the intestine, during adulthood [7], [8], [9], [10], [11], [12].

The term macrophage is derived from the Greek words *makros* and *phagein* and literally means ‘big eater’. While Mϕs are specialised in phagocytosis, this is not their only role. Rather Mϕs perform an array of functions during the innate immune response and in the initiation of inflammation, as well as contributing to tissue development, homeostasis and repair. While the precise contribution of origin to tissue-resident Mϕ function is an ongoing question, there is considerable evidence that origin may not be the deciding factor in determining function. All Mϕ progenitors were shown to be capable of generating functional lung Alveolar Mϕs (AMs) when transferred into neonatal *Csf2rb2*^-/-^ mice [13] and similarly in the liver, the transcriptional profile of monocyte-derived Kupffer cells (KCs) was highly homologous to the profile from their embryonic counterparts [8]. Thus, it seems that the local environment into which the progenitor enters decides the fate and functions of the differentiated Mϕ [7]. Crucially, Mϕs in each tissue display unique epigenetic landscapes contributing to specialised transcriptional profiles [8], [14], [15] suggesting that each tissue-resident Mϕ population will have some functions that are specific for their tissue of residence. For example, splenic red pulp macrophages are specialised in iron acquisition and metabolism [16], [17], Muscularis Mϕs in the intestine function to regulate gastrointestinal motility [18], Peritoneal Mϕs have been reported to contribute to the recruitment of B1 cells to the peritoneal cavity through their expression of CXCL13 [19], Mϕs in the bone, termed osteoclasts, are specialised in bone resorption [20] and microglia in the brain function in the synaptic ‘pruning’ crucial in the development and maintenance of the central nervous system [21]. These additional functions which benefit the organ have been dubbed ‘accessory’ functions [22]. One such accessory function of some tissue-resident macrophages is lipid metabolism. Although required by most macrophages to deal with the lipids they acquire through the phagocytosis of dying cells, it is clear from the transcriptional profiles of different tissue-resident macrophages that some, namely the AMs and KCs, are more highly specialised in this function than others [8], [15]. This is a crucial function of these Mϕs as defective or absent lipid metabolism can result in several pathologies including pulmonary alveolar proteinosis (PAP, see below) in the lung. Moreover, development of pathologies can also lead to the recruitment of inflammatory monocyte-derived Mϕs (moMϕs) which take up and subsequently need to be able to process lipids potentially having dramatic effects on disease progression and patient outcome such as in atherosclerotic plaques. Thus, in this review, we will discuss the roles of both tissue-resident and recruited moMϕs in lipid metabolism in different tissues, the factors governing lipid metabolism functions in these Mϕs and the role of lipid metabolism by macrophages in the context of disease pathogenesis. Finally, we will highlight, what we believe to be the major burning questions that remain to be addressed in this area.

## 2 Lipid metabolism

### 2.1 Lipid metabolism pathways

**Fig. 1:**
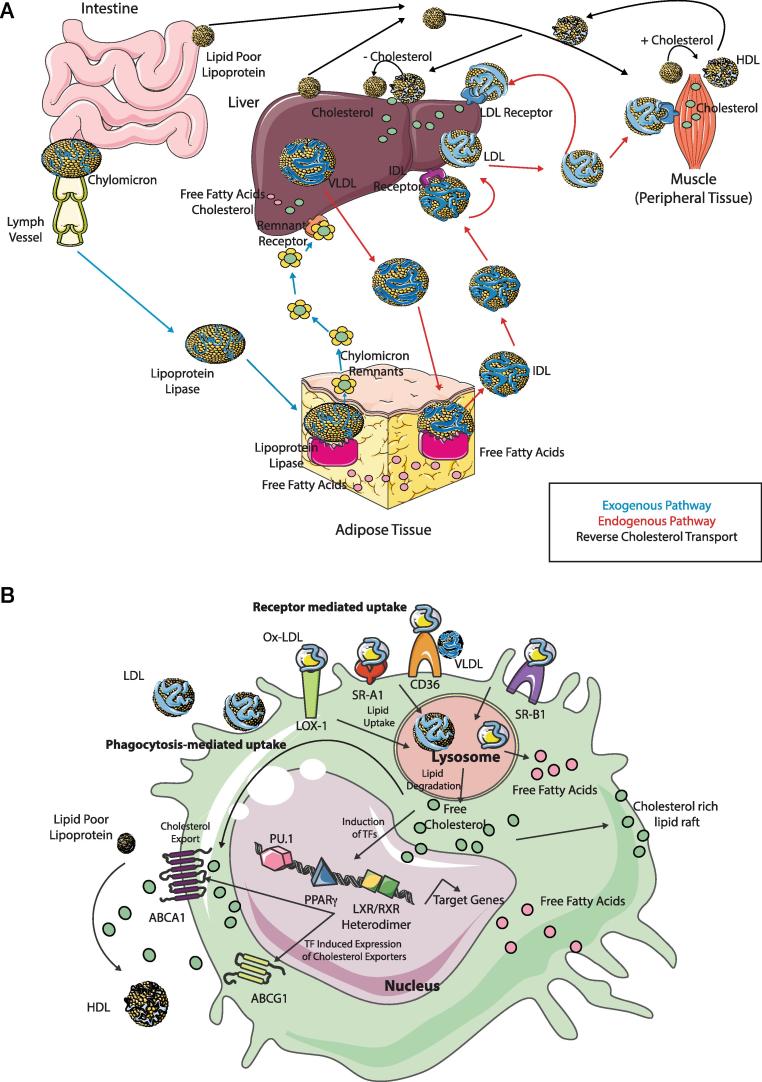
Overview of Lipid Metabolism and Macrophages. (A) The 3 pathways of Lipid Metabolism are the exogenous pathway (blue), the endogenous pathway (red) and reverse cholesterol transport (black). In the exogenous pathway, chylomicrons from the intestine are released into lymph and enter the bloodstream and subsequently adipose tissue. Here, through the action of lipoprotein lipase these are degraded into free fatty acids and chylomicron remnants which go back into circulation and then enter the liver through remnant receptors to be subsequently degraded into free fatty acids and cholesterol. In the endogenous pathway, VLDLs are exported from the liver to the circulation and adipose tissue where they are degraded again through the action of lipoprotein lipase into free fatty acids and IDL, which then binds the IDL receptor converting IDL to LDL. LDLs then bind to the LDL receptor delivering cholesterol to peripheral tissues as well as returning it to the liver. In the reverse cholesterol pathway, excess cholesterol is returned via HDL to the liver to be excreted in the bile. (B) Macrophages take up LDL, VLDL and oxidised lipoproteins via macropinocytosis, phagocytosis and scavenger receptor-mediated pathways including LOX-1, SR-A1, CD36 and SR-B1. Free cholesterol and fatty acids are generated following degradation of ingested lipids in the lysosome. Such cholesterol can be utilised to form lipid rafts. Accumulation of cellular cholesterol leads to activation of several transcription factors, including PPARγ, LXRs and RXRs which subsequently regulate expression of their target genes including transporters such as ABCA1 and ABCG1 which regulate the efflux of free cholesterol and scavenger receptors. Alternatively, passive efflux of free cholesterol can also occur. (For interpretation of the references to colour in this figure legend, the reader is referred to the web version of this article.)

The primary function of lipid metabolism is to deliver lipids to peripheral tissues for use or to return lipids to the liver for recycling or clearance. There are 3 main pathways of lipid metabolism: exogenous, endogenous and reverse cholesterol transport. The exogenous pathway is used to process dietary lipids. The endogenous pathway refers to the processing of lipids synthesised in the liver and the process of removing cholesterol from tissues and returning it to the liver is termed reverse cholesterol transport. These pathways are summarised in Fig. 1A. In the exogenous pathway, digested dietary fats; triglycerides (TGs) and cholesterol are packaged into chylomicrons in intestine epithelial cells. These packages are then released into the lymphatic system, acquire Apolipoprotein (Apo)B and subsequently get into the circulation. In the circulation, they acquire ApoC-II, ApoC-III and ApoE at different concentrations [23]. ApoC-II is recognised by adipose tissue where lipoprotein lipase (LPL) hydrolyses TGs releasing free fatty acids for cellular uptake and releasing chylomicron remnants into the bloodstream. These chylomicron remnants are removed from the plasma through remnant receptors on the liver [24]. In the endogenous pathway, the liver synthesises very low-density lipoprotein (VLDL) packages containing TGs and cholesterol assembled with ApoB. VLDLs are hydrolysed in fat tissue by LPL into VLDL remnants (or IDLs) and free fatty acids for cellular uptake. IDLs are then hydrolysed by hepatic lipases to low density lipoproteins (LDLs). LDLs transport most cholesterol. Binding of LDLs to their receptors (LDLRs) results in their uptake and release of free cholesterol. Remaining LDLs can be bound by free ApoA secreted by the liver forming Lipoprotein A. This can then bind to the extracellular matrix thus being deposited in vessels leading to atherosclerosis under conditions of excess LDL [25].

Reverse cholesterol transport then returns cholesterol to the liver. This is crucial for homeostasis as most cells in peripheral organs cannot catabolize cholesterol [26]. The liver and intestines also secrete lipid poor ApoA-I. This is lipidated in the circulation via cholesterol efflux by transporters located in peripheral tissues and macrophages [27]. Acquiring cholesterol generates mature high-density lipoproteins (HDLs). Cholesterol is then selectively removed from the particle by the liver. Excess cholesterol is then excreted into the bile and lipid poor HDL is hydrolysed or returned to circulation for relipidation [23], [27] (Fig. 1A).

### 2.2 Macrophages and reverse cholesterol transport

The same mechanisms that have evolved to enable macrophages to protect the body from infection, such as phagocytosis of pathogens, also renders macrophages key players in lipid metabolism. As macrophages readily take up lipoproteins from dying cells, they have also evolved mechanisms for eliminating cholesterol from the cell. If excess cholesterol is not eliminated from macrophages, this leads to the generation of foam cells. Such foam cells are a contributing factor in the development and rupture of atherosclerotic plaques [28] (see below). Macrophages possess 4 distinct pathways for exporting free cholesterol to HDLs; 2 passive and 2 active [26]. Macrophages take up LDL, VLDL and oxidised lipoproteins, via macropinocytosis, phagocytosis and scavenger receptor-mediated pathways (Fig. 1B) [29]. Ingested lipids are digested in the lysosome, generating free cholesterol and free fatty acids. Free cholesterol can then be re-esterified in the endoplasmic reticulum (ER) to cholesterol fatty acid esters enabling it to be stored in the cytosol in lipid droplets generating the ‘foam’ of foam cells (Fig. 1B) [30]. Alternatively, free cholesterol can be effluxed from the cell at the plasma membrane. Accumulation of cellular cholesterol leads to activation of several transcription factors (TFs) (see below) including the liver X receptor α and β (*Lxra*, *Lxrb*), the retinoid x receptor (*Rxr*) and members of the peroxisome proliferator-activated receptor (Ppar) family including *Ppara* and *Pparg* [31], [32]. *Lxr* and *Rxr* form heterodimers and these TFs upregulate the expression of the ATP-binding cassette subfamily A member 1 (ABCA1) and ABCG1 [33], [34], [35]. These transporters regulate the efflux of free cholesterol to lipid poor ApoA-I and other poorly lipidated apolipoproteins to eventually form mature HDLs [36] (Fig. 1B), although the precise contribution of ABCG1 *in vivo* remains controversial [33], [37], [38]. Alternatively, passive efflux of free cholesterol can also occur either via simple diffusion (aqueous diffusion pathway) or facilitated diffusion (SR-BI-mediated pathway) (Fig. 1B) [26].

### 2.3 Fatty acid oxidation

Lipid metabolism results in the generation of free fatty acids that are subsequently taken up by different cells. Additionally, as described above, macrophages can acquire lipids through scavenger receptors such as CD36 on their surface. Such lipids are then degraded in the lysosome via the action of lysosomal acid lipase into free cholesterol and fatty acids, in a process termed lipolysis [39]. While cholesterol is exported from the cell to HDLs, the fatty acid oxidation (FAO) pathway enables the fatty acids to subsequently be converted in the mitochondria into numerous products that the cell can use to generate energy such as acetyl-coenzyme A, NADH and FADH_2_. Initially fatty acids in the cytosol are ‘activated’ via an enzyme-mediated reaction with ATP to generate fatty acid acyl-CoA [40]. These then enter the mitochondria either via passive diffusion (short-chain fatty acids) or via the carnitine shuttle whereby medium/long chain fatty acids are conjugated to carnitine via the enzyme activity of carnitine palmitoyl transferase 1A (CPT1A) and transported into the mitochondria. Once there, CPT2 removes the carnitine and β -oxidation of the fatty acid acyl-CoA occurs [40], [41]. This yields large amounts of acetyl-CoA, NADH and FADH_2_ that are subsequently used in the TCA cycle and electron transport chain to generate ATP.

### 2.4 Fatty acid synthesis

For a cell to grow and proliferate lipids are required. If the lipid levels in the cell are not sufficient then the fatty acid synthesis (FAS) pathway can be initiated in the cytoplasm to allow cells to generate lipids from precursors derived from other cell intrinsic metabolic pathways including the TCA cycle, glycolysis and the pentose-phosphate pathway [40], [41]. mTOR signalling promotes FAS through the induction of sterol regulatory element binding protein (SREBP) a transcription factor (see below) which in turn induces fatty acid synthase (FASN) and acetyl CoA carboxylase (ACC) [42].

## 3 Macrophage function and fatty acid metabolism

As mentioned above, recent studies have highlighted that the local environment in which the macrophages reside rather than their origin likely governs their function [8], [13]. In terms of function, macrophages are often divided into discrete subsets termed M1 or classically activated and M2 or alternatively activated macrophages. M1 macrophages are characterised by high levels of pro-inflammatory cytokines and, because of their substantial production of reactive oxygen and nitrogen species, are highly microbicidal. Consistent with this they also promote T_h_1 type responses. Conversely M2 macrophages are described as predominantly anti-inflammatory and are associated with wound healing, tumour growth, helminth infections and T_h_2 type responses. Importantly, this classification has been made based on *in vitro* studies and recent work highlighting the heterogeneity amongst *in vivo* tissue-resident macrophage populations has demonstrated that this classification of macrophage subtypes is over-simplistic [43]. Nonetheless, while *in vivo* macrophages likely represent a spectrum between the defined M1 and M2 phenotypes *in vitro*, this classification has allowed significant advances to be made in understanding the metabolic programming of the different macrophage functions. There is accumulating evidence that different metabolic pathways are required for programming M1 and M2 Mϕs (for reviews see [40], [41], [44], [45]), Here, we will focus on the role played by FAO and FAS in Mϕ polarization.

There appears to be a reciprocal relationship between the differentiation of M1 and M2 Mϕs and their requirement for FAS and FAO respectively. Inflammatory signals including LPS and IFNγ, that are required to generate M1 Mϕs, have been shown to drive FAS [46], [47], while the inhibition of inflammatory signals required for the differentiation of M2 Mϕs involves FAO [40], [48], [49]. Intriguingly, cellular longevity is also thought to be supported by FAO [50], [51], thus it is tempting to speculate that this may also be true in tissue-resident macrophages which are renowned as long-lived self-renewing cells (Reviewed in [2], [52]), however this remains to be examined. The IL4 induced M2 phenotype is dependent upon signal transducer and activator of transcription 6 (STAT6) and the peroxisome proliferator-activated receptor γ (PPARγ) and its coactivator 1b (PGC1b) [53], [54]. Moreover, epigenetic reprogramming through the activity of demethylating enzymes such as JMJD3 plays a role in M2 polarization [45], [55], [56]. Inhibition of FAO, using pharmacological inhibitors such as Etomoxir, prevents M2 activation and overexpression of PGC1b prevents an M1 response following stimulation with LPS and IFNγ [39], [53]. The source of the fatty acids enabling FAO in M2 Mϕs has been shown to derive from uptake via the CD36 receptor (which is induced by IL4) and subsequent lysosomal lipolysis mediated by lysosomal acid lipase (which can be further induced by IL4) [39]. Moreover, fatty acids fueling FAO can be generated through glycolytic metabolism as M2 Mϕs consume more glucose and glutamine than naïve Mϕs and glutamine deprivation has been shown to impair M2 polarization [57], [58], [59], [60]. Consistent with this, it has recently been reported that α -ketoglutarate generated from glutaminolysis can promote M2 activation by augmented FAO via Jmjd3-dependent metabolic and epigenetic reprograming [60]. However, the precise role of FAO in driving M2 polarization is currently under scrutiny as genetic ablation of *Cpt2* (the enzyme which removes carnithine allowing β-oxidation of the fatty acid in the mitochondria to occur), in Mϕs using LysM^CRE^xCpt2^fl/fl^ mice does not impair M2 polarization in bone marrow-derived Mϕs or peritoneal Mϕs [61]. The discrepancy between the data using Etomoxir or genetic ablation of *Cpt2* could be ascribed to off target effects of Etomoxir which is thought to inhibit CPT1a (which transports long-chain fatty acids in the mitochondria) or a role for CPT1a outside of FAO but further work is required to determine this. Alternatively, this finding may represent a shortcoming of the LysM CRE transgenic mouse model, which does not efficiently target all Mϕs. Importantly, while the exact role of FAO in inducing the M2 phenotype requires further study, increasing FAO in lipid laden foam cells such as those observed in atherosclerosis (see below) by enforcing CPT1a expression can reduce lipid accumulation as well as the production of pro-inflammatory cytokines [62] suggesting that inducing FAO in foam cells could be of therapeutic potential.

## 4 Transcription factors modulating lipid metabolism activity in Macrophages

While the M1/M2 classification holds *in vitro*, the situation is more complex *in vivo*. In recent years, it has become clear that different tissue-resident Mϕ populations have distinct transcriptional, epigenetic and metabolic profiles [8], [14], [15], [44], [45]. These profiles arise as a result of the complex and distinct myriad of signals these Mϕs receive in each tissue. These tissue-specific signals also impart ‘so-called’ accessory functions on these Mϕ populations [22], [63], that the Mϕs must perform in addition to more general Mϕ functions such as phagocytosis. Although all Mϕs take up and process lipids to some extent, lipid metabolism could be viewed as one such accessory function of Mϕs as the transcription profiles of lung alveolar Mϕs (AMs) and liver Kupffer cells (KCs) are both enriched for this function compared with other Mϕ populations [8], [15]. Several transcription factors (TFs) are associated with the specific accessory functions of Mϕs. In terms of lipid metabolism, such TFs expressed by the different Mϕ populations include the Liver-X family of receptors (LXRs), Peroxisome proliferator-activated family of receptors (PPARs), CCAAT enhancer binding proteins (C/EBPs) and sterol regulatory element binding proteins (SREBPs) (See Table 1). In addition, although not a TF, Micro-RNA 33 (miR-33) has also been implicated in regulating lipid metabolism in macrophages and hence will also be discussed here.

**Table 1:** Overview of TFs modulating lipid metabolism in macrophages.

TF	Role(s) in lipid metabolism	Role(s) in macrophages	Reference(s)
LXRs	Cholesterol sensors Reverse cholesterol transport	Expressed by Splenic marginal zone Mϕs and Kupffer cells Reduce cellular cholesterol levels Modulation of glucose metabolism Limiting the production of inflammatory mediators Regulates presence of splenic marginal zone Mϕs	65–75, 79, 80
PPARγ	Required for adipocyte differentiation Regulates expression of scavenger receptors involved in lipid uptake Regulates expression of cholesterol efflux genes (with LXRα)	Expressed by Alveolar Mϕs, Kupffer cells and Splenic Red Pulp Mϕs, Required for Alveolar Mϕ development & maintenance Implicated in M2 polarization Inhibits pro-inflammatory gene expression Required for apoptotic cell engulfment	82–92
C/EBPβ	Adipose tissue development Regulation of glucose and lipid metabolism Regulation of b-oxidation & lipogenic genes & fatty acid synthase	Expressed by most Mϕs Induced in human Mϕs by OxLDLDrives M2 macrophage polarization Loss of C/EBPβ results in an altered phenotype of alveolar Mϕs and large peritoneal Mϕs	93–96,100
SREBPs	Regulators of cholesterol and lipid synthesis Induces LDLR expression	Induced by NF-kB activation Regulates lipogenesis Drives Inflammasome activation	101

The liver X receptors (LXRα and LXRβ, encoded by *Nr1h3* and *Nr1h2* respectively), form part of the nuclear receptor family of TFs. LXRs bind to DNA as heterodimers with RXRs and as such the activity of the heterodimer can be modulated by ligands of either partner. LXRβ is relatively ubiquitously expressed while LXR α is more restricted in its expression being found predominantly in metabolic tissues such as the liver and adipose tissue and in tissue-resident macrophages [64]. More specifically, LXRα is highly expressed in splenic macrophages and liver Kupffer cells [8], [14], [15]. This is reflected in the gene expression profiles of these cells which include numerous LXRα target genes including *Cd5l* [8], [15]. Analysis of F4/80, CD68 and CD169 expression in LXRα/β double KO mice revealed that LXRs are required for presence of splenic marginal zone macrophages but not other macrophages including liver Kupffer cells (at least based on the expression of these common Mϕ surface markers) despite their high expression of these TFs [65]. The main function of LXRs is as cholesterol sensors, regulating gene expression in response to specific oxysterol ligands [66]. In macrophages, such oxysterols may be derived from internalized oxLDL or generated intracellularly through modification of cholesterol [67], [68]. Perhaps the best-known function of LXRs is in the regulation of reverse cholesterol transport (see above). In macrophages, LXRs reduce cellular cholesterol levels by regulating the expression of the cholesterol efflux transporters ABCA1 and ABCG1 [69], [70], [71], [72], [73]. In addition to inducing expression of cholesterol efflux transporters, LXR signalling can induce the expression of apolipoproteins such as ApoE and ApoC which serve as receptors for cholesterol [74], [75]. The importance of apolipoproteins in macrophage cholesterol efflux is evident as mice lacking ApoE specifically in macrophages are more susceptible to atherosclerosis [76]. Increased production of lipoprotein remodeling proteins including LPL is a further mechanism by which LXRs function in macrophages to reduce the cholesterol burden [77]. Finally, it has also been reported that LXR induced expression of Idol in macrophages can induce LDLR ubiquitination leading to its degradation via the proteasome and subsequently reduced LDL cholesterol binding and uptake [78]. In addition to reducing cholesterol levels, several studies have also implicated LXRs in the modulation of glucose metabolism and in innate and adaptive immune responses. In macrophages, the latter equates to LXR activation limiting the production of inflammatory mediators such as iNOS and Cox2 [79], [80].

The peroxisome proliferator-activated receptors (PPARs) family of transcription factors are a nuclear hormone receptor superfamily, having established roles in lipid metabolism. PPARs, like LXRs are active when heterodimerised with RXRs [81]. The family includes PPARα, associated with hepatic and cardiac lipid metabolism, the ubiquitously expressed PPARβ (also known as PPARδ) and PPARγ, which is highly expressed in adipose tissue, where it is essential for adipocyte differentiation [82], and macrophages and hence will be the focus of this review. PPARγ is highly expressed in AMs in the lung [15], [83], [84]. Here it is induced by CSF-2 signalling and is required for AM development and maintenance [84], [85]. It is also expressed (albeit at lower levels) by splenic red pulp Mϕs and Kupffer cells [8], [84], although the role of PPARγ in these cells under homeostatic conditions remains unknown. In addition to being expressed by these tissue-resident Mϕ populations, it has been reported that PPARγ expression is upregulated in Ly6C^hi^ monocyte derived Mϕs recruited to the peritoneum following thioglycollate treatment [84]. Functionally, PPARγ has been suggested to control the inflammatory potential of Mϕs being implicated in driving M2 polarization of Mϕs as well as inhibiting pro-inflammatory gene expression including IL1 β, IL6, TNF α, IL12 and iNOS [54], [86], [87]. This is fitting with results showing that if PPARγ is lost from Mϕs recruited to the peritoneum following thioglycollate treatment, resolution of inflammation is impaired [84]. However, more recently PPARγ has also been shown to be required for uptake of apoptotic cells by Mϕs [88], providing an alternative mechanism through which PPARγ in Mϕs contributes to the resolution of inflammation. In addition, perhaps the best-known role of the fatty acid receptor, PPARγ, is in cholesterol metabolism. PPARγ has been shown to regulate lipid accumulation in Mϕs in atherosclerotic plaques. It achieves this by regulating expression of the scavenger receptors involved in lipid uptake, CD36 and SR-A [86], [89], as well as (through activating LXRα) regulating expression of cholesterol efflux genes including the transporters ABCA1 and ABCG1 [90], [91] (see above). As a result of this function, low or absent expression of PPARγ in Mϕs is associated with increased atherosclerosis [90], [92] and insulin resistance [54].

The C/EBP family of TFs are basic region-leucine zipper (bZIP) proteins. This family of proteins have been associated with adipose tissue development [93], regulation of glucose and lipid metabolism [94] as well as M2 Mϕ polarization [95]. Although the roles of C/EBPs in lipid metabolism in Mϕs are not as well characterised as PPARs and LXRs, we are beginning to understand the role played by one family member C/EBPβ. In terms of tissue-specificity amongst Mϕs, C/EBPβ is expressed by most tissue-resident Mϕ populations [15]. However, to date it has only been suggested to be required for the presence of lung AMs and large peritoneal Mϕs (LPMs), as C/EBPβ ^-/-^ mice appear to lack both of these populations on the basis of their expression of a number of typical surface markers [96]. Notably, C/EBPβ is also required for the differentiation of Ly6C^lo^ monocytes from their Ly6C^hi^ counterparts [97], a process crucial in the pathogenesis of atherosclerosis (see below). Mice lacking C/EBPβ are completely protected against diet induced obesity with reduced expression of hepatic lipogenic genes, fatty acid synthase and increased expression of β-oxidation genes in brown adipose tissue [98]. Evidence that C/EBPβ may play a role in lipid metabolism in Mϕs comes from studies showing that C/EBPβ expression is induced in human Mϕs primed with OxLDL to become foam cells alongside LXRα and PPARγ expression [99]. Furthermore, ChIP-Seq analysis for C/EBPβ in these Ox-LDL exposed human Mϕs found an enrichment of genes associated with both the innate immune response and lipid/cholesterol transport and efflux [99]. Likewise, in mice, C/EBPβ was also found to regulate lipid metabolism genes such as PPARγ, LXRα in peritoneal and RAW Mϕs, although the direction of this regulation was not always consistent between the different Mϕs [100]. It is also worth noting that this study preceded the one showing that LPMs were reduced or altered in the absence of C/EBPβ [96] and hence it remains to be investigated if this difference in gene expression is truly due to the loss of C/EBPβ or if it instead reflects differences in the macrophage populations present in C/EBPβ -sufficient or-deficient animals.

SREBPs encoded by *Srebf1* and *Srebf2* are master regulators of cholesterol and lipid synthesis [101]. They belong to the family of basic-helix-loop-helix leucine zipper TFs. Intracellular cholesterol, desmosterol and oxysterols retain SREBPs in the endoplasmic reticulum, however when levels drop, SREBPs can enter the nucleus, bind to sterol regulatory elements (SREs) and induce gene transcription inducing cholesterol and fatty acid synthesis [44]. Accumulation of newly synthesized sterols then prevents further SREBPs from being cleaved and exiting the ER, providing a negative feedback loop. Loss of this feedback loop through deletion of the genes which maintain SREBP in the ER (*Insig1* and *Insig2*) is detrimental, resulting in lipid accumulation which has been shown to cause lipotoxicity in alveolar macrophages [102]. SREBP1a is the main isoform found in macrophages. In addition to its role in lipid synthesis, SREBP1a also induces LDLR expression and is also associated with inflammatory function. In Mϕs, SREBP1a expression can be induced by NF-κB activation, which drives enhanced lipogenesis as well as inflammasome activation and subsequent IL1β release [103], [104]. However, SREBPs are not solely pro-inflammatory, SREBP-1 KO macrophages following TLR4 activation have been shown to be hyper-inflammatory as they are unable to synthesize anti-inflammatory unsaturated fatty acids leading to increased expression of *Il1a*, *Cxcl1* and *Cxcl9* [105]. Thus, like the other TFs discussed above, SREBPs are involved in the regulation of a myriad of processes further highlighting the importance of lipid metabolism in macrophage function.

Unlike TFs which bind to *cis*-regulatory DNA elements often located in or near their target genes, microRNAs (miRNAs) hybridize to *cis*-regulatory RNA elements mostly located in the 3′ untranslated region of their target mRNAs. In terms of regulating lipid metabolism, miR-33 and its passenger strand miR-33∗ have been shown to play a significant role [106], [107], (for review of miRNAs in lipid metabolism see [108]). miR-33 has also been implicated in determining the activation state of macrophages [109] (see below). miR-33a and miR-33b are co-expressed with their host genes *Srebf1* and *Srebf2* and their function is to prevent the initiation of pathways opposing those induced by SREBPs [106], [107]. Thus, they repress the expression of genes involved in cholesterol efflux and FAO. This pathway holds promise as a therapeutic target. Inhibitors of miR-33 result in an increase in expression of the cholesterol transporter ABCA1 in macrophages (and in the liver) and hence cholesterol efflux suggesting an anti-atherosclerotic effect [106], [107], [110], [111], [112], [113], [114], [115]. However, its potential use is broader than atherosclerosis as it has also recently been shown that *Mycobacterium tuberculosis* (*Mtb*) is capable of surviving in macrophages by inducing expression of miR-33 and miR-33∗ inhibiting autophagy, lysosomal function and FAO, the latter providing the bacteria with a source of nutrients in the form of cholesterol and fatty acids [116]. Notably, mice lacking miR-33 in hematopoietic cells had an improved ability to clear the bacteria, while treatment of macrophages *in vitro* with miR-33 and miR-33∗ inhibitors also restricted *Mtb* viability [116].

Taken together, it is clear that the presence or absence of lipids can have dramatic effects on macrophage biology, affecting their gene expression profile and ultimately their functions. Thus, it is no surprise that macrophages have been implicated in the pathogenesis of several diseases in which lipid homeostasis is perturbed such as atherosclerosis, non-alcoholic fatty liver disease (NAFLD) and alveolar proteinosis. In the next section, we will review the roles played by different macrophages in some of these lipid-mediated diseases.

### 4.1 Atherosclerosis

**Fig. 2:**
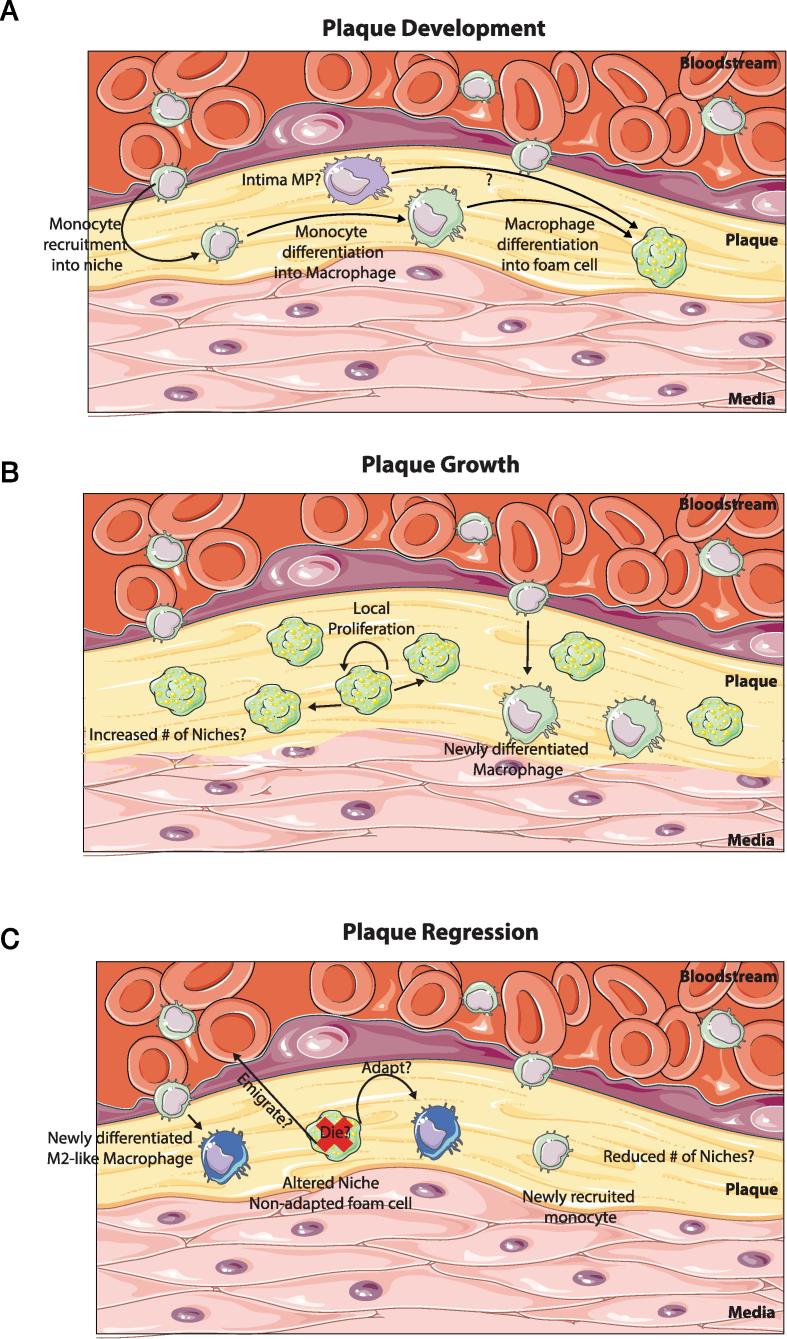
Macrophage Niches in Atherosclerosis. (A) The onset of atherosclerosis is associated with plaque development. This generates new macrophage niches which need to be filled. This is done predominantly through the recruitment of monocytes to the developing plaque and their subsequent differentiation into macrophages. These macrophages then take up the excess fat and differentiate into lipid laden foam cells. (B) As the plaque grows, likely so too does the number of macrophage niches. These are then populated through 2 main mechanisms. The predominating mechanism is proliferation. Foam cells already present in the plaque sense the empty niches and start to proliferate thus filling up the available niches. Alternatively, new monocytes can also be recruited to the growing plaque where they will engraft and then differentiate into macrophages. (C) During plaque regression, the local environment is changed dramatically. This can have two consequences, the foam cells which are adapted to the fat-rich environment cannot adjust to the new environment and subsequently die or possibly emigrate out, resulting in niche availability which is filled through monocyte recruitment and subsequent differentiation into macrophages that are distinct from those generated in the fat-rich environment being more M2-like. Alternatively, if plastic enough to do so, the foam cell may alter its transcriptional profile rendering it more suited to this new fat-poor environment. Additionally, as the plaque regresses it is likely that the number of Mϕ niches decreases.

Atherosclerosis, the leading cause of death in industrialized countries, is a chronic inflammatory disease. It arises as a result of an accumulation of cholesterol-laden macrophages in the artery wall leading to an imbalance in lipid metabolism and a maladaptive immune response [28]. Atherosclerosis develops when excess cholesterol-rich apolipoprotein B, in the form of LDLs and remnants accumulate along the endothelium. This accumulation forms plaques in the subendothelium of the arteries. This occurs in regions where laminar flow is disturbed or insufficient such as at curves and branching points in the vessels [28]. Such accumulation of lipid leads to the recruitment of monocytes into the subendothelial space, which subsequently differentiate into macrophages with a predominantly pro-inflammatory phenotype (Fig. 2). The metabolic factors driving the macrophage phenotype in atherosclerosis have recently been reviewed by Stienstra and colleagues [44] and Bories and Letinger [117] and hence will not be discussed further here. Recruitment of monocytes is proportional to disease burden and hypercholesterolemia results in monocytosis through the regulation of stem cell cycling by cholesterol in the membrane [118], [119], [120]. Evidence from the clinic suggests that this is also true in humans with low HDL correlating with increased monocyte numbers [121], [122]. Monocytes can be recruited from the BM or from a splenic reservoir [123]. There are 2 subsets of monocytes in the blood, namely Ly6C^hi^ and Ly6C^lo^ in mice and CD14^+^ and CD16^+^ in humans [124]. The Ly6C^hi^/CD14^+^ subset constitutes the majority of cells recruited to progressing plaques and hence these are thought to be the main source of the macrophages that develop in the plaque [118], [119]. While Ly6C^lo^ monocytes are hypothesised to play a protective role (reviewed in [125]), the mechanisms through which monocytes are recruited to plaques have been extensively reviewed elsewhere and so will not be discussed here (for examples see [126], [127], [128]). In addition to monocyte-derived macrophages, macrophage-like cells derived from smooth muscle cells also accumulate in the plaque [129], [130], [131], [132]. Furthermore, low numbers of mononuclear phagocytes (MPs) are present in the intima which may also contribute to macrophage burden in plaques, especially as lipid-laden foam cells are detected in within the first week following feeding of a cholesterol-rich diet to Ldlr^-/-^ mice [133], a time before a significant increase in monocyte recruitment is detected [134]. These cells have been identified in the intima as early as the 4th week of life [135], but earlier time-points remain to be examined. Thus it is interesting to speculate that these MPs may represent embryonically-derived tissue-resident macrophages, especially given that while Ly6C^hi^ monocytes are recruited at low levels [134], [136], there is little evidence to suggest they give rise to the MPs in the intima prior to the onset of atherosclerosis. However, the true identity of these cells remains controversial as they appear to share features of the both the macrophage and dendritic cell lineages, thus further study is required to accurately determine the nature of these cells (reviewed in [125], [137]). The exact roles of the distinct lineages of macrophages in atherosclerosis remain to be investigated (Box 1). As a result of these unknowns, in the rest of this section, we will discuss the role of macrophages in general in atherosclerosis.

Box 1 **Macrophage Niches in the Atherosclerotic Plaque**

The macrophage niche hypothesis was recently proposed enabling, in our opinion, the current, seemingly conflicting data on macrophage ontogeny to be reconciled [7]. This model predicts the ontogeny of different macrophage populations based on three main factors, niche availability, niche accessibility and precursor plasticity [7]. Although at this time purely speculative, it is tempting to apply the same concept to the atherosclerotic plaque (Fig. 2). The plaque could house novel niches for macrophages upon its development. Alternatively, the niches could exist prior to the onset of atherosclerosis and expand in number (and possibly be altered due to the inflammation) during atherosclerosis. Indeed a minor population of MPs are present in the intima, although whether these are *bona fide* macrophages is still debated [137]). At the onset of atherosclerotic plaque formation, Mϕ numbers are dramatically increased. One of the signals potentially driving this is cholesterol as hypercholesteraemia correlates with monocytosis [118], [119], [120]. The increased numbers of macrophages could derive from 3 potential sources. As the niche is accessible [7], being in the artery wall, one method of increasing Mϕ number is through the recruitment of monocytes to the plaque which enter the subendothelial space and differentiate into Mϕs. Ly6C^hi^ monocytes have been shown to be the main contributors to the Mϕ pool [210], however, the development of atherosclerosis is only halted when recruitment of both monocyte subsets (Ly6C^hi^ and Ly6C^lo^) is abolished through the inhibition of CCR5, CX3CR1 and CCL2 in the *Apoe*^-/-^ model [119], [211]. This could suggest that both monocyte subtypes are recruited and give rise to the eventual foam cells. In addition to monocyte-derived Mϕs, smooth muscle cells (SMCs) have been shown to transdifferentiate into Mϕ -like cells thus also potentially contributing to Mϕ numbers [130], [132], [212]. Finally, proliferation of the resident MP population likely also contributes to the increased Mϕ number in the developing plaque as foam cells are present prior to the recruitment of monocytes upon switching to a cholesterol rich diet [133], [134], however, whether these cells are maintained and proliferate in the progressing plaque remains unclear. Typically, inflammation induces death of resident macrophage populations, the so-called ‘macrophage disappearance reaction’. As resident macrophages, that have been conditioned for prolonged periods of time in one niche, appear to have limited plasticity when transplanted into a second different niche [13], [14], it is plausible that the resident intima MPs, see the increased lipid levels, try to deal with the lipid but fail resulting in their death and subsequently most Mϕs in the developing plaque derive from monocytes or SMCs. Importantly, the different contributions of the distinct Mϕ populations to the pathophysiology of atherosclerosis remain unknown. Based on recent data from other tissues [8], [13], one could hypothesise that if the local signals driving the differentiation of monocyte-derived and SMC-derived Mϕ populations in the developing plaque are the same then their functions could also be similar. Of course, all this will depend upon the plasticity of both the monocytes and SMCs as well as the putative long-lived resident Mϕ population, which remains to be investigated. After developing, the atherosclerotic plaque itself is a dynamic structure. As the plaque grows, this could be envisaged as an increase in the number of niches resulting in niche availability. This availability is subsequently filled via two mechanisms. Macrophages already present in a niche proliferate to fill new niches while (albeit at a lower level) new monocytes are also recruited to fill the new niches [138], [213]. This is consistent with open niches such as the liver, whereby Kupffer cells, following partial depletion, are repopulated through both local proliferation and monocyte recruitment and differentiation [8]. In addition to plaque growth creating niche availability, lipid-laden foam cells also routinely die in the plaques thus again creating niche availability which can also be filled through both mechanisms of repopulation. During plaque regression the niche hypothesis could also potentially be applied. If one considers that conditioned foam cells are plastic, then these may adapt to the new environment becoming more M2-like. However, if the foam cells are not particularly plastic and hence cannot readily adapt to the new environment, (dramatic lipid lowering seen in regression), the Mϕs would not be able to adapt and would subsequently die, or potentially emigrate out (discussed in [28], [125]) once again creating niche availability. This availability would then be filled by recruited monocytes which would then differentiate into macrophages adapted to the new niches. For example, in the model of atherosclerotic plaque transplantation into WT mice, the rapid reversal of dyslipidaemia drives the replacement of the more M1-like macrophages in the niches with newly recruited Ly6C^hi^ monocytes which differentiate into more M2-like macrophages [169]. Importantly, at least in this model, regression requires the *de novo* recruitment of Ly6C^hi^ monocytes and their subsequent differentiation as blocking monocyte recruitment prevents regression from occurring [169].

Macrophages build up in the developing plaques and are maintained predominantly through proliferation with limited input from monocytes [138] (Box 1, Fig. 2). In the plaque, macrophages acquire lipoproteins from the surrounding environment. This can occur through the scavenger receptors (pattern recognition receptors; PRRs) which are present on the cell surface of the macrophages. Multiple types of these scavenger receptors are present including scavenger receptor A1 (SR-A1), MARCO, CD36, SR-B1 and LOX1 which take up oxidised forms of LDL (ox-LDL) generated as a result of the increased oxidative stress in the artery wall (reviewed in [139], [140]). Notably, as PRRs, these scavenger receptors do not only function in lipid uptake. For example, SR-A1 has also been implicated in modulating macrophage proliferation within the lesion, thus also regulating macrophage numbers [138]. CD36 has been implicated in inflammasome activation [141] and macrophage polarization [142] and both of these receptors are also linked to promoting apoptosis and inflammatory gene expression [143]. Alternatively, LDL, when present at hyperlipidemic concentrations, can be engulfed by macrophages via pinocytosis [144] and lipolytic enzymes present in the intima can also generate modified forms of LDL that are taken up by macrophages using scavenger receptor independent pathways [145]. Under normal conditions, lipids taken up by macrophages are typically processed and effluxed from the cells preventing the generation of foam cells (described above). However, as there appears to be little negative feedback, during dyslipidaemia excessive lipid uptake by macrophages results in defective cholesterol trafficking and lipid efflux and the generation of foam cells engorged with lipid [28], affecting macrophage phenotype and compromising immune functions.

Free cholesterol is toxic to cells, as a result free cholesterol is typically transported to the ER where it is re-esterified to form cholesterol esters that can be stored as relatively inert material in lipid droplets or effluxed from the cell via transporters such as ABCA1. Excess free cholesterol in the ER, for example due to impaired efflux, can result in defective re-esterification promoting further accumulation of free cholesterol in the cell, such as at lipid rafts in the membrane [146]. This causes enhanced inflammatory signalling at the membrane lipid rafts including TLR signalling and NFκB activation [146], [147], [148]. Inflammatory signalling is further exacerbated if cholesterol trafficking from lysosomes becomes defective. Combined, these phenomena contribute to increased ER stress in the macrophages which results in cell death by apoptosis [149], [150], [151], [152], [153], [154]. Apoptotic macrophages must be cleared by other macrophages in the vicinity by efferocytosis. However, this requires intact lipid metabolism. In atherosclerosis, the dysregulated lipid metabolism in macrophages prevents effective efferocytosis and this coupled with the increase in macrophage apoptosis results in secondary necrosis [155], [156]. One mechanism for this is the cleavage of the efferocytosis receptor MerTK from macrophages by ADAM17 [157], [158], [159], [160], [161]. The subsequent release of intracellular components into the local environment contributes to the establishment of a necrotic core in the plaque. This is a feature of advanced atherosclerotic plaques and can contribute to plaque rupture. Moreover, through the secretion of matrix metalloproteinases, macrophages are thought to further contribute to plaque rupture [162], however as mouse models do not exhibit similar rupture as observed in humans this has been difficult to validate experimentally.

While associated with plaque development, progression and rupture, macrophages are also implicated in plaque regression suggesting that macrophages may also play atheroprotective roles. Regression can be induced experimentally through drastic lipid reduction or surgical models including transplantation of an aortic plaque into a WT mouse with normal lipid levels [110], [163], [164], [165], [166], [167]. Macrophage content is typically reduced during regression (Fig. 2), however, the phenotype of the macrophages in the plaque is also altered, adopting a more M2-like phenotype. Macrophages in the regressing plaque express elevated levels of *Arg1* and *Cd163* and reduced levels of pro-inflammatory genes including *Tnfa* [168]. This altered gene expression profile is not however black and white M2 vs M1 as seen *in vitro*, as some inflammatory genes are upregulated during regression including *Il1b* [168]*,* further highlighting the shortcomings of this classification *in vivo* [43]. Additionally, it remains controversial if this change in gene expression profile represents a shift in macrophage phenotype or recruitment of new cells [125], however, the latter concept is supported by recent data showing monocytes are indeed recruited to regressing plaques where they differentiate into M2-like macrophages [169], [170] fitting with the recently proposed macrophage niche hypothesis [7] (Box 1, Fig. 2). Taken together, it is evident that macrophages through their roles in both lipid metabolism and the immune response are central to the pathophysiology of atherosclerosis. Given that dysregulated lipid metabolism is the driving factor in foam cell development, macrophage activation and subsequent plaque rupture resulting from development of the necrotic core, further understanding the factors that affect the lipid content of macrophages and their responses to such lipid will be key in developing therapeutic interventions.

### 4.2 Non-Alcoholic fatty liver disease

Non-alcoholic fatty liver disease (NAFLD) represents a spectrum of liver diseases including non-alcoholic fatty liver (NAFL, isolated steatosis,) non-alcoholic steatohepatitis (NASH), fibrosis, cirrhosis and hepatocellular carcinoma (HCC). Global prevalence of NAFLD ranges from 22 to 28% [171] and is currently increasing linked to the increase in obesity and type 2 diabetes [172]. The pathogenesis of NAFLD remains incompletely understood but the multiple hit theory is commonly accepted, whereby parallel hits from the gut and adipose tissue drive hepatic fat accumulation and inflammation [173]. Hepatocellular accumulation of lipids is a key event in the early stages of NAFLD while progression to NASH is fuelled by hepatic inflammation. Hepatic Mϕs are thought to play a key role in this progression [174]. As discussed in more detail below, this role is thought to be primarily due to their modulation of hepatic inflammation through their response to DAMPs and other signals released as a consequence of increased hepatic fat burden [175]. However, it is worth noting that as KCs possess all the genetic machinery to take up and metabolize excess lipids [8], their involvement in NASH pathogenesis may also be related to lipid accumulation and processing. Indeed Leroux and colleagues have shown that specific lipids accumulate in KCs of mice fed a high fat diet (HFD) and that this results in altered expression of lipid metabolism associated genes [176]. Thus, this avenue of study requires further investigation.

NAFLD is commonly associated with a pro-inflammatory hepatic Mϕ phenotype. This is primarily associated with the presence of DAMPs and PAMPs such as LPS, FFA and cholesterol and the release of additional DAMPs by dying fat-laden hepatocytes which trigger Mϕ activation. In addition to these DAMPs and PAMPs, a recent study has shown that hepatocyte-derived histidine rich glycoproteins (HRG) can promote M1 responses in Mϕs *in vitro* and that HRG^-/-^ mice display attenuated liver injury and fibrosis on the methionine-choline deficient diet (MCDD), a murine model of NASH [177]. Interestingly, this attenuation also correlated with decreased hepatic Mϕ numbers and a more M2-like phenotype in those remaining compared with WT controls [177]. Hepatocyte HRG was also found to be significantly upregulated in NAFLD patients and this correlated with a M1-like phenotype of adjacent Mϕs [177] suggesting HRG is another factor driving the pro-inflammatory phenotype in hepatic Mϕs during NAFLD. Toxic lipid accumulation in Mϕs was also associated with a pro-inflammatory phenotype suggesting that altered lipid metabolism may also contribute to the role of KCs in modulating hepatic inflammation in NAFLD [176]. Additionally, another recent study has shown that hepatocytes triggered *in vitro* by fatty acids release extracellular vesicles (EVs) containing tumour necrosis factor related apoptosis inducing factor (TRAIL) in a Rho-associated, coiled-coil-containing protein kinase 1 (ROCK1) dependent manner and that these EVs induce the production of *Il1b* and *Il6* mRNA in murine bone-marrow derived Mϕs [178]. Importantly, ROCK1 inhibition *in vivo* in a model of NASH reduced liver injury, inflammation and fibrosis [178]. While most research suggests pro-inflammatory Mϕs dominate in NAFLD and drive disease pathogenesis, hepatic Mϕs may also have anti-inflammatory properties. Moreover, promoting such M2-like Mϕs has been reported to be beneficial for protecting against NAFLD [177], [179], [180], [181], [182], [183]. Taken together, these results suggest that manipulation of the M1/M2 ratio in NAFLD may be an attractive therapeutic strategy [184].

**Fig. 3:**
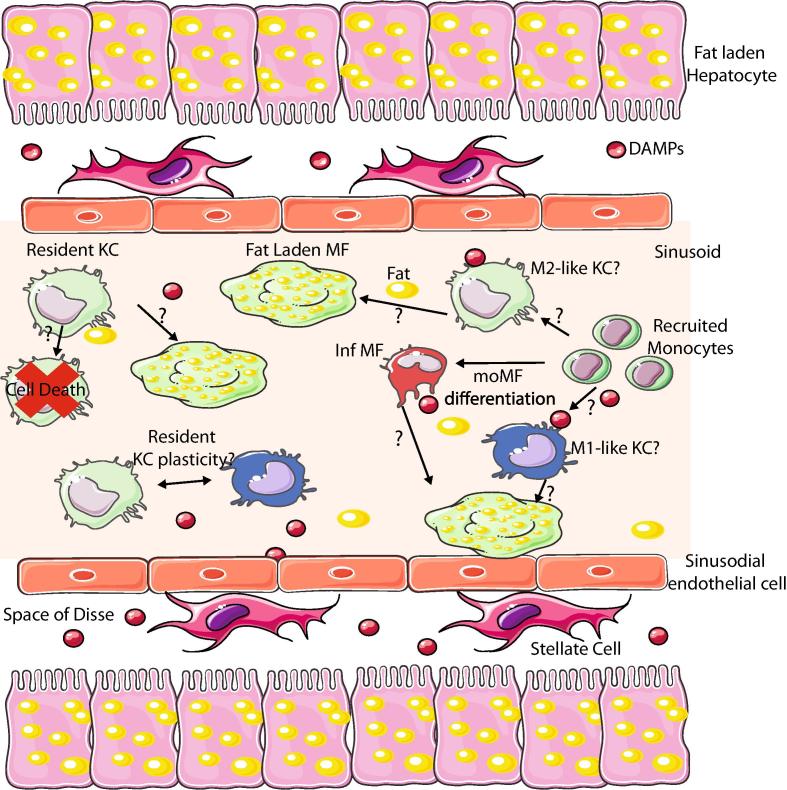
Role of Mϕs in NAFLD. There are several questions remaining regarding the role of Mϕs in NAFLD. One of the main questions relates to whether there are multiple subsets of KCs and other hepatic Mϕs present or not. Monocytes are recruited to the liver and develop into infiltrating Mϕs (Inf Mϕs) during NAFLD, but do they also contribute to the *bona fide* KC pool? If so are these moKCs more M1 or M2-like? Another question is where do the foam cells originate from? KCs that have become overloaded with lipid, or can these cells given their lipid metabolism enriched transcriptional profile efficiently process lipids meaning the foam cells are generated from the infiltrating Mϕs or is it a combination of both? Another key question is whether KCs are plastic and can change from a more M2 profile to that of an M1-like Mϕs and vice versa. If this is the case, how does this work and can it be manipulated to enhance M2-like cell numbers given that this appears beneficial for the patient?

While there are numerous studies suggesting that modulating the M1/M2 balance may be beneficial in NAFLD, it remains unclear if M1 and M2 macrophages represent discrete Mϕ populations or in fact if one Mϕ population expresses both M1 and M2 genes that can be further skewed in one direction or another. As mentioned above, the M1/M2 classification does not really hold *in vivo* given the myriad of signals present at any one time [43], thus the true nature of these cells remains largely unknown. Related to this, until recently it has been difficult to distinguish between *bona fide* Kupffer cells and other hepatic Mϕs that may be recruited during NAFLD. This has made it difficult to assess the role of each Mϕ subset in NAFLD. Indeed most strategies to look at KC function target both KCs and recruited hepatic Mϕs including LysM Transgenic mice and Clodronate Liposomes, the latter of which targets all phagocytic cells, making results difficult to interpret [185], [186], [187]. In addition, multiple studies have shown recently that monocytes can also give rise to *bona fide* KCs [8], [12], [14] suggesting that the hepatic Mϕ pool during NAFLD could consist of at least 3 distinct populations. While all of these express high levels of F4/80, CD64 and lack Ly6C expression, they can be distinguished on the basis of Clec4F and Tim4 expression [8]. Those Mϕs expressing Clec4F^+^Tim4^+^, the long-lived KCs, the Clec4F^+^Tim4^-^ monocyte-derived KCs and Clec4F^-^ hepatic Mϕs [8]. CX3CR1 and CCR2 are two other markers which can be used to identify hepatic Mϕs which have recently derived from monocytes [188], although they do not distinguish between monocyte-derived KCs or other Mϕs. While these populations were first identified in livers under predominantly homeostatic conditions, we have recently also shown that all three populations can be identified during NASH in mice fed a MCDD [189] and during feeding of a high fat, high cholesterol and high sugar diet (our own unpublished observations). Thus, now it will be necessary to determine the functional contributions of each of these populations in disease pathogenesis. A number of other questions also remain (Fig. 3). As described for atherosclerosis, one crucial question lies in the plasticity of hepatic Mϕs [7]. Do long-term resident KCs dramatically alter their gene expression profiles upon exposure to excess fat or other signals, such as increased LPS? Lipid metabolism has been implicated in macrophage polarization but is it enough to alter a cell that has been programmed for prolonged periods in conditions without excess fat? There is no clear consensus as to whether resident Mϕs would be plastic or not, AMs in the lung do seem to alter their transcriptional programmes during fibrosis [190], however following paracetamol overdose, KCs have been reported to have very few changes in their gene expression profile [188], thus further investigation is warranted. Other questions include; do KCs die during NAFLD? What role do the infiltrating Mϕs play? These differentiate amidst the myriad of danger signals, thus, are they for that reason predominantly pro-inflammatory? Indeed, these infiltrating monocyte-derived Mϕs have been suggested to play a role in disease pathogenesis as blocking/reducing their infiltration can attenuate NASH [187], [191], [192]. Do *bona fide* KCs differentiate from monocytes during NAFLD? If so, how different is their transcriptional/epigenetic profile compared with those who differentiated during homeostatic conditions? Are they also long-lived? While the majority of these questions remain unanswered, there is some recent work suggesting answers to these questions. We have found that moKCs generated during NASH appear to be relatively short lived being lost upon recovery [189] while other data suggests that infiltrating monocytes may indeed have a distinct phenotype and possibly function compared with the resident KCs [187], however, the gating strategy used in this study to identify the different subsets may have led to some overlap and so further investigation using new markers is required. Crucially, we now have the tools to address these questions, meaning exciting times lie ahead for our understanding of hepatic Mϕ involvement in NAFLD.

### 4.3 Macular degeneration

The inability to efficiently efflux cholesterol does not only play a role in obesity-related diseases. Age related macular degeneration (AMD), the leading cause of blindness in the industrialised world in the ageing population [193], is another disease where impaired cholesterol efflux has been implicated in the pathophysiology [194], [195], [196]. AMD occurs in two forms early (or dry) and exudative (wet). Blindness largely occurs from the wet form of AMD which is characterised by development of new blood vessels underneath the retina, termed choroidal neovascularization (CNV) [197]. Wet AMD is preceded by dry AMD. Dry AMD is characterised by the presence of esterified-cholesterol rich ApoB lipoprotein laden deposits called ‘Drusen’ in a layer of the retina termed Bruchs membrane. Increased size and number of Drusen is a major factor involved in AMD progression [198]. Moreover, Drusen, like atherosclerotic plaques, result in monocyte recruitment, macrophage differentiation and associated inflammation [199]. Macrophages have been implicated in all stages of AMD. While macrophages recruited during early stages of disease have a predominantly pro-inflammatory (M1-like) phenotype, AMD progression and CNV development, in both mice and men, is associated with a pro-angiogenic (M2-like) macrophage phenotype [197], [200], [201], [202], [203]. Macrophages sample Drusen and subsequently process and export the cholesterol. It has recently been shown that impaired cholesterol efflux in macrophages due to loss of *Abca1* expression can also lead to a pro-angiogenic phenotype and thus contribute to CNV development [194]. Interestingly, *Abca1* expression is downregulated in senescent macrophages, which possess a predominantly pro-angiogenic phenotype [194]. Thus, highlighting the need for efficient cholesterol efflux by macrophages not only in obesity-driven diseases but also in limiting AMD progression.

### 4.4 Pulmonary alveolar proteinosis

**Fig. 4:**
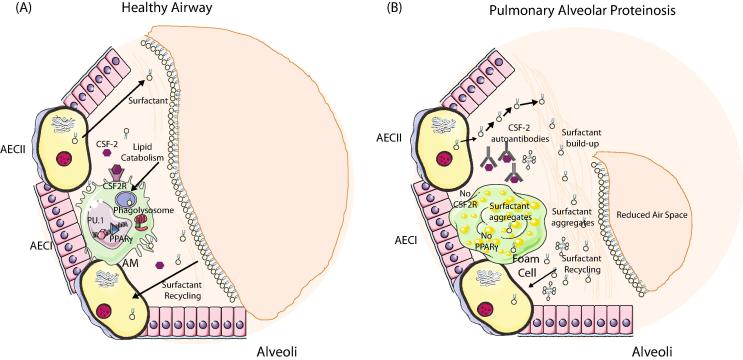
Role of Mϕs in Alveolar Proteinosis. (A) Normal alveolar macrophage development occurs within the first week of life. Local CSF-2 signalling in the alveoli induces expression of the TF *Pparg* in the CSF-2R expressing fetal liver monocytes generating pre-alveolar macrophages, which subsequently differentiate into AMs. Type II alveolar epithelial cells (AECII) produce surfactant that is crucial for normal lung function. Excess or old surfactant must be recycled and this occurs primarily via the AECII but also via AMs. Surfactant is taken up via the AMs and catabolised in the phagolysosome. (B) In the absence CSF2 signalling as a result of deficiency in CSF2, CSF2R or the presence of auto-antibodies directed against CSF2, *Pparg* expression is not induced and as a result AMs do not develop correctly and cannot process the excess lipids. This leads to a build-up of surfactant in the alveoli and macrophages, the latter of which results in the generation of foam cells. This build up leads to a reduction in the air space in the alveoli making it difficult to breathe.

Although not involved in metabolism of cholesterol, alveolar macrophages of the lung also play a role in lipid metabolism. Pulmonary alveolar proteinosis (PAP) is a rare disease characterised by build-up of surfactant within the alveoli [204]. Pulmonary surfactant is produced by type II alveolar epithelial cells (AEC) and is essential for normal lung function. It forms mono and multi-layers that act as the interface between liquid and air, reducing surface tension and allowing efficacious gas exchange. It also contributes to host defence acting as an opsonin as well as directly killing microbes [205]. Normal levels of surfactant, which is composed of phospholipids and proteins, are crucial and regulated through its balanced production, secretion, uptake, recycling and catabolism [205]. 70% of spent surfactant is taken up by type II AECs and recycled while the remaining 30% is taken up and catabolized by AMs. This function of AMs is crucial as evidenced by the development of hereditary PAP in patients and mice lacking AMs due to deficiencies in either CSF2 (GMCSF) or *Csf2r* [206], [207] and idiopathic PAP in patients with non-functional AMs due to neutralizing auto-antibodies against CSF2 [208]. CSF2 signalling is crucial for the development of AMs and hence the acquisition of this surfactant catabolism function [6] (Fig. 4). Transcriptional profiling of AMs has shown that they possess a strong lipid metabolism signature within their core genes [15], [84]. One way through which CSF2 signalling drives AM development is through the induction of the transcription factor *Pparg* which is crucial for AMs with *Pparg* conditional knock out mice lacking functional AMs [84], [85] (Fig. 4). In the absence of CSF2 signalling or *Pparg* expression F4/80^+^CD64^+^ Mϕs are found in the lung alveolar space but these do not express SiglecF or CD11c, two characteristic markers of AMs [6], [84], [85]. These Mϕs are hence not *bona fide* AMs and as such lack specific AM-associated functions including the ability to catabolise surfactant. This results in the build-up of surfactant within the Mϕs leading to the generation of foam cells with dramatically altered gene expression profiles [84], [204], and, a build-up within the alveoli leading to respiratory difficulties (Fig. 4). Transplantation of AMs as well as *Csf2rb2*-sufficient Mϕ progenitors (fetal liver monocytes, yolk-sac Mϕs and BM monocytes) has been shown to be sufficient to prevent PAP development in *Csf2rb2*^-/-^ mice [13], [209], further highlighting the essential role played by AMs in PAP progression and providing a potential new therapy option for this disease.

## 5 Conclusions and future perspectives

In conclusion, it is evident that macrophages play key roles in lipid metabolism and in the pathogenesis of lipid-related diseases. However, it is also clear that we do not yet fully understand this. There are a number of outstanding questions in the field (see above and summarised in Box 2). Thus, we still have much to learn regarding the fascinating roles of macrophages particularly in lipid metabolism. Importantly, as our understanding of macrophages increases, both in terms of their common and tissue-specific features, we are more and more in a position to design new tools to address such specific questions and as such exciting times lie ahead in terms for macrophage biology.

Box 2 **Burning Questions.**

While it is obvious from the literature reviewed here that Mϕs play predominant roles in lipid metabolism both in the steady state (AMs) and during disease pathogenesis (Atherosclerosis, NAFLD, AMD & PAP), it is also very clear that we do not yet fully understand this and as a result many pertinent questions remain to be answered by the field. For example, with the addition of recent data the role of lipid metabolism in driving Mϕ phenotype is being debated. Thus, the question remains what role does lipid metabolism play in regulating the phenotype of Mϕs *in vivo*? Moreover, it will be important to understand how lipid metabolism is regulated in Mϕs? It has been reported that senescent Mϕs downregulate their expression of cholesterol efflux genes. What regulates this? Furthermore, how does this translate in terms of lipid load in macrophages or the tissues in which they reside?. In addition to this, another question that remains unanswered is why is the transcriptional profile of certain Mϕs enriched for lipid metabolism genes compared with others? While for some this is clear (AMs and surfactant clearance), the role(s) of the other Mϕs enriched for this function, such as the KCs, is/are largely unknown. Are these cells contributing to lipid clearance and recycling similar to their neighbours, the hepatocytes? Moreover, how are these cells then involved in conditions of excess lipid such as NAFLD? Are they solely driving NAFLD pathogenesis by acting as sensors of DAMPs and PAMPs? Or does their role go beyond this into lipid uptake and clearance? More importantly, is this function conserved in human KCs? And can manipulating the KCs in terms of their lipid metabolism function alter and ultimately improve patient outcome? This is something we are actively investigating in the lab. Another key and perhaps the most crucial question for the field, is the question of Mϕ subsets and plasticity. Many studies have reported the presence of M1 Mϕs at one stage of disease and M2 Mϕs at another. However, two things remain unclear with this characterisation. Firstly, the relevance of the use of the M1/M2 nomenclature *in vivo.* We know that the M1/M2 classification does not hold up *in vivo* and as often these Mϕs are defined on the basis of one or two surface markers it is really unclear what these cells actually are. Secondly, does this reflect truly different subsets of macrophages at different disease stages or macrophage plasticity? Are these distinct subsets of Mϕs recruited at different stages of the disease? Perhaps resident versus recruited Mϕs? Or is it one subset of cells that changes it function along a spectrum based on the signals around it? How plastic are Mϕs? Do they adapt to changes in local lipid level or do they die and get replaced? With the so-called Mϕ disappearance reaction occurring during most insults, one might consider that resident Mϕs are not actually that plastic and hence once their environment or niche is altered they cannot adjust and hence die being replaced by monocyte-derived cells, but this remains to be investigated. This also then leads to the question, how plastic are the recruited monocytes? Are they altering their function during disease progression? Or are they again replaced by a more adapted Mϕ when the environment changes?
